# Return to Play in Long-Standing Adductor-Related Groin Pain: A Delphi Study Among Experts

**DOI:** 10.1186/s40798-021-00400-z

**Published:** 2022-01-18

**Authors:** Luca Vergani, Marco Cuniberti, Massimo Zanovello, Daniele Maffei, Abdulaziz Farooq, Cristiano Eirale

**Affiliations:** 1 Università degli Studi di Siena, Siena, Italy; 2 Aspetar Orthopaedic and Sports Medicine Hospital, Doha, Qatar; 3 FC Lugano, Lugano, Switzerland; 4 Bergamo Basket 2014, Bergamo, Italy; 5 Paris St Germain FC, Paris, France

**Keywords:** Long-standing groin pain, Sports injuries, Return to play, Delphi

## Abstract

**Background:**

Groin pain is a very common injury in multidirectional sports such as soccer, ice hockey, rugby and Australian football. Long-standing adductor-related groin pain is a persistent clinical condition and a frequent complaint in athletes involved in sports that require multiplanar movement patterns (change of direction, high-speed sprinting and kicking). To date, the lack of rehabilitation guidelines and return-to-play criteria makes this clinical entity difficult to manage. The aim of the present Delphi was to suggest, based on opinion and practical experience of a panel of experts, potential criteria that could be taken into consideration by clinicians in the RTP decision-making process in athletes suffering from long-standing adductor-related groin pain.

**Methods:**

Thirty two out of 40 experts participated to a 3-Round Delphi questionnaire. In round 1, open-ended and closed questions about 9 different sections (palpation, flexibility, strength, patient-reported outcome measures, imaging, intersegmental control, performance tests, sport-specific skills, training load) were proposed to investigate return to play evaluation criteria used by each expert. Responses were analysed and coded to produce round 2 questionnaire that investigated only the sections and the items that reached the cut-off value (≥ 70%). Round 3 questionnaire was based on sections and items that reached cut-off value in previous rounds and experts rated their agreement for return to play criteria with a 5-point Likert Scale. Descriptive statistics enabled interpretation of consensus.

**Results:**

High participation rate (80%) and response rate across the 3 rounds (100%) were recorded. 6 sections reached positive consensus in round 1, 1 section reached negative consensus. In round 2 positive consensus was confirmed only for 3 sections and negative consensus for 1 section. In round 3, positive agreement was established for strength (3 items), performance tests (3 items) and sport-specific skills (2 items) sections. Negative consensus was confirmed for imaging section.

**Conclusion:**

Experts agreed that strength, performance tests and sport-specific skills can be used to support RTP decision, while imaging cannot be used. These findings could be useful in assisting clinicians in the RTP decision making.

**Supplementary Information:**

The online version contains supplementary material available at 10.1186/s40798-021-00400-z.

## Key Points

- Strength assessment, performance tests analysis and sport specific skills evaluation can be considered helpful in RTP assessment in athletes with long-standing adductor-related groin pain (LARGP).
- Imaging methods are not considered useful in RTP decision making process.
- Agreement established between experts in groin pain can assist clinicians in RTP decision making.

## Background

Groin pain provides a massive challenge for all those involved in diagnostic, rehabilitation and physical preparation of athletes at all levels due to the complex anatomy of the groin region and the poor understanding of the adverse mechanisms that predispose the athlete to injury [1, 2].

Studies in professional sports have found groin injury to be the fourth most common injury in soccer [3] and the third most common injury in Australian rules football [4]; it has also shown to have a high prevalence in ice hockey [5] and rugby [6].

Long-standing adductor-related groin pain (LARGP) is a persistent clinical condition with gradual or sudden onset characterised by adductor tenderness and pain on resisted adduction testing [7]. It is a frequent complaint in athletes involved in multidirectional field sports that require multiplanar movement patterns, such as change of direction (COD) [8, 9], high-speed sprinting [4, 10] and kicking [11].

In accordance with Strategic Assessment of Risk and Risk Tolerance (StARRT) framework [12, 13], return to play (RTP) decision making is a complex process based on the evaluation of health and activity risks but it is also influenced by the assessment of the risk tolerance modifiers.

Combining information from biological, psychological and social standpoints can help all RTP decision-makers (clinician, physiotherapist, coach) to make optimal and shared decisions [14].

Nevertheless, RTP criteria for many common injuries like groin pain are not based on solid scientific evidence due to the lack of clarity and consensus on the term ‘return to play’ [14]. So far, no studies have specified which criteria should be assessed by clinicians to allow an athlete suffering from groin pain a timely and fully RTP.

The aims of this Delphi study were to reach an agreement between a panel of experts, based on opinion and practical experience, and suggest potential criteria that could be taken into consideration by clinicians in the RTP decision-making process in athletes suffering from LARGP.

## Methods

### Purpose and Rationale

The Delphi is a group facilitation technique that seeks to obtain consensus on the opinion of “experts” through a series of structured questionnaires commonly referred to as “rounds” [15]. The Delphi is therefore an interactive multistage process designed to combine opinion into group consensus [16, 17]. The initial questionnaire may also collect qualitative comments which are feedback to the participants in a quantitative form through a second questionnaire [15].

This scientific method has been effectively used in Sports Medicine research [18–21].

The whole process lasted from February 2020 to July 2020. A total of 3 rounds were carried out using the platform https://www.google.com/intl/it/forms/about/.

### Steering Committee

The Delphi survey was created by a 5-member steering committee consisting of four sports physiotherapists and one sport physician, all with background in clinical research and elite sport.

### Expert Panel and Procedure

In accordance to previously Delphi studies published [21–23], to be considered eligible, to participate in the study, only healthcare practitioners meeting the following inclusion criteria were deemed eligible: (1) 2 or more peer-reviewed publications in the field of groin pain in athletes and (2) experience in scientific methodology and/or (3) clinical expert and designated member of the conference organising committee and (4) follow evidence to guide their clinical decisions and (5) sufficient knowledge of the English language.

According to “snowballing method” [24], each expert contacted could, in turn, invite 3 additional experts then submitted to inclusion criteria.

The experts were contacted (26 directly invited, a further 14 suggested by experts; *n* = 40) via e-mail and they were asked to be willing to participate in the study and information about the aim and methodology of the study were provided. Participants were given 1 month to complete the questionnaire in each round, with email reminders sent to non-responders after 10 days and 20 days, respectively.

### Round 1

Round 1 was the only round prepared before the beginning of the study because each subsequent round was dependent on the responses from the previous one.

Written explanation of the experimental procedure was provided to each individual; this included the aims of the study, the experimental procedures to be utilised and a clear explanation of the use of the definition of LARGP [7] and the use of the definition of RTP [25]. Individuals then provided written, informed consent before participating in the study.

The first round was divided into 2 parts. The first one investigated the “demographic” characteristics of the participants: profession, affiliation, years of experience in the field of sports medicine, the number of athletes treated with groin pain/year, the number of peer-reviewed studies published around groin pain (Table 1).

**Table 1:** Demographic characteristics of participants

Number of participants	32 (100%)
Region	
Europe	28 (87.5)
USA	1 (3.1)
Australia	3 (9.4)
Affiliation	
Clinical	24 (75.0)
Academic	6 (18.8)
Team	2 (6.3)
Profession	
Physiotherapist	21 (65.6)
Physiologist	1 (3.1)
Physician	5 (15.6)
Surgeon	5 (15.6)
Mean ± SD	
Experience (years)	20.8 ± 10.0
Peer review publications on groin pain in last 10 years	11.7 ± 12.4
Peer review publications on groin pain in total	15.7 ± 22.1

The second part included 38 questions divided into 9 different sections (palpation, flexibility, strength, patient-reported outcome measures (PROMs), imaging, intersegmental control, performance tests, sport specific skills, training load). All sections were selected based on the literature, with the objective of investigating the clinical assessment of each researcher in the evaluation of RTP.

During the first round both close-ended and open-ended questions were used.

The sentence “Do you use/analyse “X” when evaluating RTP in LARGP?” was the first closed-ended question put at the beginning of each section.

Within the section there were also open-ended questions to provide the researcher the possibility to motivate his answer and/or indicate aspects not considered in the question asked.

In accordance with Joyner et al. [26], the answers to each open-ended question were divided into categories. In order to reduce categorisation bias responses were independently coded by 2 different researchers (MZ and MC), and compared only at the end to discuss the final categorization [20]. The 3 categories with the highest consensus were then included in the second round and submitted to other researchers [26].

### Round 2

At the beginning of the second round, the categories that reached the cut-off value were listed and the aim of the study was explained again. Round 2 questionnaire investigated only the categories that reached the cut-off value. The first question in each section asked whether or not the researcher considered the category concerned as a RTP criterion. The following questions were formulated based on the answer given within the round 1 questionnaire, feedbacks and suggestions in order to go into more details surrounding each of the categories.

### Round 3

Round 3 questionnaire investigated only the answers that have reached the cut-off value in round 2.

For all items that reached the cut-off value in round 2, researchers were asked to express their degree of consensus by using the Likert-scale [27] with values from 1 to 5 (Strongly Disagree, Disagree, Neutral, Agree, Strongly Agree).

At the end, participants were given the opportunity to share comments on the whole Delphi process.

## Data Analysis

Data from all Delphi rounds were collected using Google online forms and extracted to IBM SPSS V.21 for statistical analysis. Two of the steering committee members independently performed content analyses and a third investigator was consulted whenever there were any disagreements/ambiguity around the tagging, categorising and interpreting the responses. In closed-ended questions (option yes/no or specific items from a list to be selected), the frequency of each expert’s response was recorded and converted to a percentage (%). For open-ended questions, following recommendations by Côté et al. [28], qualitative data (ie, expert answers, justifications and suggestions) were coded, listed and compared in order to produce clusters of similar concepts which adequately represent the information received by experts. If responses to analogies reached ≥ 70% threshold [18, 20, 23, 29, 30] that particular item/criterion was considered as reaching consensus among the experts and was thereafter retained and elaborated on in further rounds, while those concepts not reaching consensus were discarded. Content analysis was used throughout rounds 1 and 2. Regarding round 3, ratings for each item coded (1–5) were expressed as means with standard deviation (SD). Consensus between participants was measured using coefficient of variation (CV%) and percentage agreement (%AGR) [31]: CV% is a measure of dispersion and %AGR was defined as the percentage of responses falling within the top two categories of the 5-point scale (Agree and Strongly agree).

Agreement between participants was also evaluated across all items using Kendall’s W coefficient (W) of concordance, a non-parametrical statistic that is used to assess strength and changes of agreement between raters [31]. In round 3, Mean rating ≥ 3.5, CV% ≤ 30%, %AGR ≥ 70% and W < 0.05 were defined as concurrent requirements for consensus in order to define a final agreement between experts for RTP in LARGP. Statistical significance was set at p < 0.05.

## Results

32 experts over 40 (80%) accepted the invitation to participate in the study and the response rate across the three rounds was 100%.

On the 9 different criteria proposed for RTP, full consensus was achieved on strength, performance tests, sport-specific skills (positive agreement) and imaging (negative agreement).

### Round 1

The sections that reached positive consensus in round 1 were: Palpation (78%), Strength (97%), PROMs (72%), Intersegmental Control (72%), Performance tests (78%), Sport-specific skills (87.5%).

The section Imaging reached a negative consensus (75%), however, the sections Flexibility and Training Load did not reach any consensus.

As reported in Table 2, consensus was achieved by 1 item (1/2) in the Palpation section, 5 items (5/21) in the Strength section, 2 items (2/8) in Intersegmental Control, 2 items (2/8) in Performance tests and 2 items (2/2) in Sport-specific skills.

**Table 2:** Expert panel answers in round 1

Section	Item	Consensus	Percentage (%)
Palpation	Use of palpation in RTP process	+	78.1
	Presence of pain in palpation	+	92.0
	Allow RTP with pain in palpation	NC	56.5
	Other parameters considered^a^	Answered by 25 experts	
	Pain parameters^b^	7 Respondents	
	Localization^b^	5 Respondents	
	Tightness^b^	4 Respondents	
	Type of tissue^b^	4 Respondents	
Flexibility	Flexibility analysis in RTP process	NC	62.5
Strength	Strength analysis in RTP process	+	96.9
	Hip muscle groups (type of strength)		
	Adductors (Ecc/Iso/Con)	+Ecc; +Iso	87.1/74.2/38.7
	Abductors (Ecc/Iso/Con)	NC	51.6/51.6/12.9
	Extensors (Ecc/Iso/Con)	NC	38.7/9.7/16.1
	Flexors (Ecc/Iso/Con)	NC	54.8/32.3/29.0
	Internal rotators (Ecc/Iso/Con)	NC	38.7/9.7/22.6
	External rotators (Ecc/Iso/Con)	NC	38.7/6.5/19.4
	Strength assessment of other muscle groups	+	71.0
	Which other areas?^a^	Answered by 22 experts	
	Trunk group^b^	18 Respondents	
	Knee group^b^	9 Respondents	
	Calf complex group^b^	7 Respondents	
	Presence of pain in strength tests	+	96.8
	Allow RTP with pain in strength tests	−	70.0
	Other parameters considered^a^	Answered by 31 experts	
	Pain parameters (location, grading)^b^	10 Respondents	
	Side-to-side comparison^b^	8 Respondents	
	Ratios^b^	8 Respondents	
	Baseline data^b^	7 Respondents	
PROMs	Use of PROMs in RTP process	+	71.9
	Which PROMs?^a^	Answered by 23 experts	
	Hip and Groin Outcome Score (HAGOS)^b^	21 Respondents	
	Visual Analog Scale (VAS)^b^	2 Respondents	
	Internal PROMs^b^	2 Respondents	
Imaging	Use of imaging in RTP process	−	75.0
Intersegmental control (IC)	IC tasks analysis in RTP process	+	71.9
	Which IC tasks?^a^	Answered by 23 experts	
	Single leg squat^b^	18 Respondents	
	Squat^b^	15 Respondents	
	Lunge^b^	13 Respondents	
	Presence of pain in IC tasks	+	82.6
	Allow RTP with pain in IC tasks	−	84.2
	Other parameters considered^a^	Answered by 23 experts	
	Quality of movement^b^	10 Respondents	
	Side-to-side symmetry^b^	3 Respondents	
	Pain parameters (location, grading)^b^	2 Respondents	
Performance tests	Performance tests analysis in RTP process	+	78.1
	Which performance tests?^a^	Answered by 25 experts	
	Planned/unplanned COD (45-90-180)^b^	18 Respondents	
	T-test^b^	17 Respondents	
	Illinois test ^b^	13 Respondents	
	Presence of pain in performance tests	+	100
	Allow RTP with pain in performance tests	−	80.0
	Other parameters considered^a^	Answered by 25 experts	
	Performance/intensity^b^	8 Respondents	
	Grading of pain^b^	6 Respondents	
	Movement control^b^	4 Respondents	
Sport-specific skills	Sport-specific skills analysis in RTP process	+	87.5
	Presence of pain in sport-specific skills	+	89.3
	Allow RTP with pain in sport-specific skills	−	84.0
	Other parameters considered^a^	Answered by 28 experts	
	Pain parameters (location, grading)^b^	7 Respondents	
	Performance/intensity^b^	5 Respondents	
	Specific tests^b^	3 Respondents	
	Quality of movement^b^	3 Respondents	
	Athlete feedback^b^	3 Respondents	
Training load	Internal load monitoring in RTP process	NC	56.3
	External load monitoring in RTP process	NC	59.4

+ , positive consensus; − , negative consensus; NC, no consensus; Ecc, eccentric; Iso, isometric; Conc, concentric; PROMs, patient reported outcome measures; CoD, changes of direction

^a^Open-ended question

^b^Top 3 ranked preferences for open-ended question coded independently by 2 researchers

Furthermore, Table 2 contains the items list (Top3 approved by 2 researchers through independent coding, based on participants’ answers and suggestions. The list was included in the round 2 questionnaire.

Items of sections that achieved negative or no consensus were not added to the round 2.

However, all the items included in the round 1 are available on the attachment of Additional file 1.

### Round 2

In round 2, 4 out of the 7 sections reached consensus as RTP criteria while the other 3 sections did not reach it.

A positive consensus has been confirmed for the sections: Strength (94%), Performance tests (91%), Sport-specific skills (91%).

A negative consensus has been confirmed for Imaging (78%).

Palpation, PROMs and Intersegmental Control lost the consensus obtained in round 1.

In the section of Strength 1 item (1/29) reached consensus, in Performance tests 1 item (1/7) and in Sport-specific skills 3 items (3/5).

Percentages were described in detail in Table 3.

**Table 3:** Expert panel answers in round 2

Section	Item	Consensus	Percentage (%)
Palpation	Use as a criterion in RTP	NC	68.8
Strength	Use as a criterion in RTP	+	93.8
	Tests to evaluate hip adductors' isometric strength	No tests reached cut-off value (≥ 70%)	
	Tests to evaluate hip adductors' eccentric strength	No tests reached cut-off value (≥ 70%)	
	Analysis of strength in other muscle groups	NC	66.7
	Other parameters considered (except pain)		
	Side-to-side Symmetry	+	80.0
PROMs	Use as a criterion in RTP	NC	59.4
Imaging	Use as a criterion in RTP	−	78.1
Intersegmental control	Use as a criterion in RTP	NC	34.3
Performance tests	Use as a criterion in RTP	+	90.6
	Others parameters considered (except pain)		
	Athlete feedback	+	82.8
Sport-specific skills	Use as a criterion in RTP	+	90.6
	Others parameters considered (except pain)		
	Athlete feedback	+	86.2
	Performance in skills execution	+	75.9
	Quality of movement	+	72.4

+, positive consensus; −, negative consensus; NC, no consensus;

A list of all items and full percentages is available within the Additional file 1.

Therefore, a form with 4 sections and 11 items was finalised for round 3 (agreement round).

### Round 3

Kendall’s W was significant at 0.03 (*p* < 0.001)**.**

**Fig. 1:**
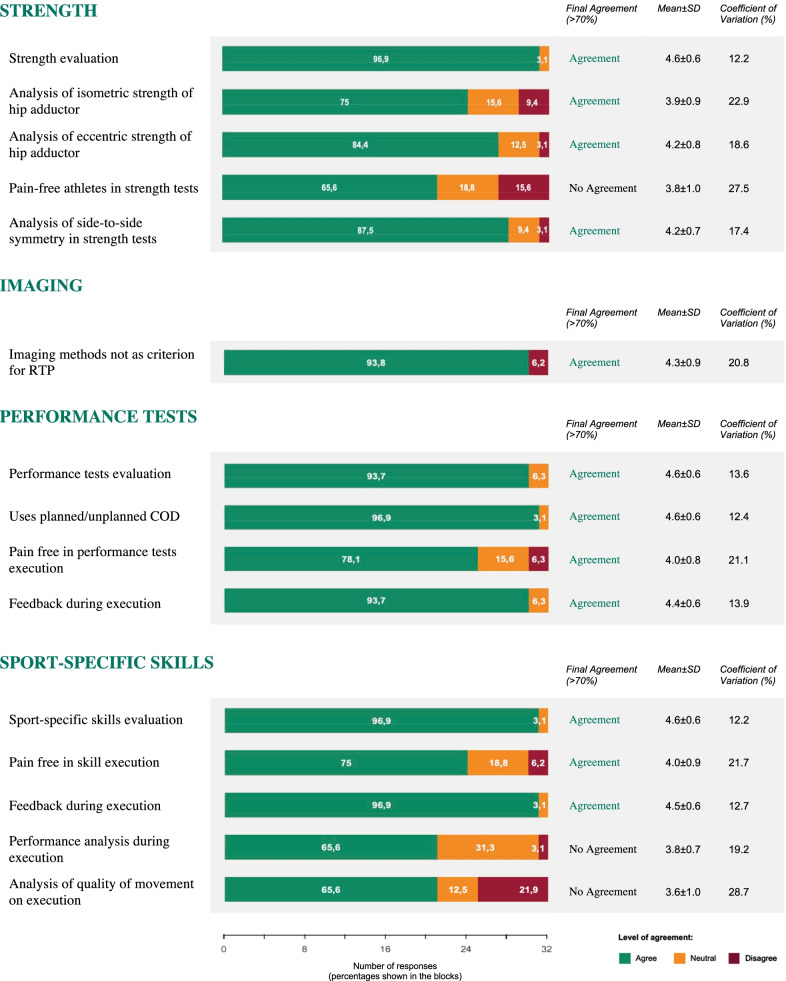
Round 3 final expert agreement on RTP criteria

Round 3 final agreement is presented in Fig. 1.

Agreement was established for the Strength section with 3 items, the Performance tests section with 3 items and the Sport-specific skills section with 2 items.

A negative consensus was established for the Imaging section.

## Discussion

The aim of this Delphi study was to achieve an agreement between experts on RTP criteria in LARGP.

The main finding was that assessment of strength, performance tests and sport-specific skills would seem to be a *sine qua non* in RTP complex process in athletes affected by LARGP.

**Fig. 2:**
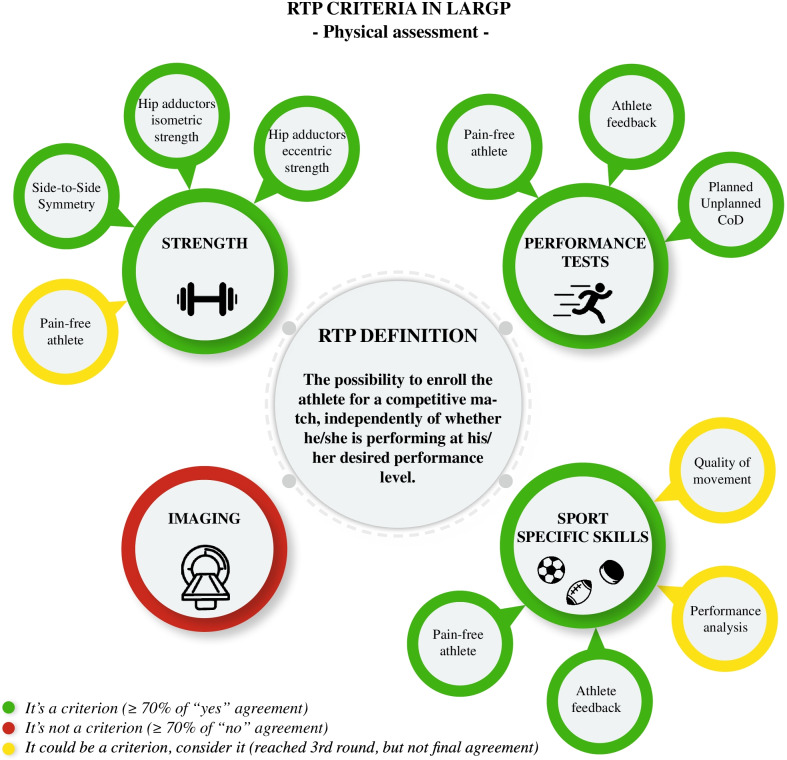
RTP criteria in long-standing adductor-related groin pain (physical assessment)

As reported in Fig. 2, it was established that during strength evaluation it would seem crucial to analyse adductors isometric and eccentric strength considering “side-to-side symmetry”.

Planned/unplanned COD analysis seems to be considered as a criterion when performance tests are evaluated; athletes should be confident during completion and totally pain free.

At the same time, during sport-specific skills analysis, athletes should be confident and completely pain free during execution.

Although few items and 4 out of 9 categories reached final agreement, low CV% (mean 18.3%, range 12.9–28.7), high %AGR (mean 84.4%, range 65.6–96.9%) and *W* = 0.03 (*p* < 0.001) show the robustness of the consensus established.

### Strength

Experts agreed on the importance of strength assessment as a RTP criterion (96.9%). Specifically, at the end of 3 rounds, consensus was achieved for the evaluation of hip adductors isometric strength (75%) and eccentric strength (84.4%).

These findings are in line with several studies [32–34] and are supported by evidence that highlights the usefulness of strength both as outcome measure [35, 36] and rehabilitation criterion in groin pain [34, 37, 38].

Despite no agreement established for strength tests to be used, the squeeze test 0° for isometric strength (66.7% of answers) and eccentric strength assessment in side-lying position (53.3%) would seem to be assessment methods with a wider consensus between experts.

Side-to-side symmetry is a discriminating factor in RTP: 87.6% of participants consider this parameter as a criterion to analyse during RTP process.

Although several studies support strength assessments of other hip muscle groups [39, 40], in our study none of these groups achieved expert consensus.

No final agreement was established for strength analysis of other muscle groups; nevertheless, trunk flexors got a high rate of positive response in round 2 (90%). A total of 18 out of the 20 participants consider strength of aforementioned muscle complex important to evaluate. This could be an interesting clinical tip to consider even though no final consensus was achieved.

### Imaging

Imaging is the only section that achieved negative consensus (93.7%). In fact, experts strongly agree to not consider or include imaging methods among RTP criteria.

Although imaging can be a valid diagnostic tool to support the clinical examination and identify red flags [41], to date no study supports its use in RTP decision. Therefore, our finding would seem in agreement with literature [42, 43].

### Performance Tests

Experts agree that analysis of performance tests can be considered as a criterion to establish RTP readiness in athletes suffering from LARGP (93.7%).

No specific test reached the 3 rounds agreement, but a strong consensus (96.9%) was achieved on the use of planned/unplanned COD to varying degrees (45°-90°-110°-180°).

Data established seems to strongly agree with the current evidence [8, 44, 45]; COD is considered an evocative and provocative movement in groin pain [46, 47] and both a sport-specific movement and a reliable outcome measure [48, 49].

Experts agree that athletes must be fully asymptomatic (78.1%) and confident (93.7%) during COD execution. This seems to be confirmed by Serner et al. [50] that used COD, absence of symptoms and athlete confidence among RTP criteria, even if their study was on acute adductor injuries.

### Sport-specific Skills

To date, no study in the literature thoroughly examined the use of sport-specific skills in RTP in LARGP.

However, skills such as “kicking a ball” are considered potential causes of groin pain onset [51].

Buckthorpe et al. [52] recommended the analysis of sport-specific movements to allow the athletes a full and safe RTS.

In the present study, the sport-specific skills section achieved solid consensus (96.9%). In addition, experts agree that athletes must be asymptomatic (75%) and self-confident (96.9%) during the execution of sport-specific tasks.

Even if parameters such as quality of movement and performance in skills execution did not reach agreement, the percentage obtained among participants (65.6%) suggests that these aspects could play a role as well.

### No Agreement Sections

Three categories that did not achieve consensus (palpation, PROMs and intersegmental control) would seem to be in some way relevant in RTP decision-making although they are not considered as criteria.

It was established 78.1% of experts use palpation in RTP stage but just 68.8% of them uses it as criterion. Despite literature seeming to agree in assuming that pain-free palpation is important during RTP when considering other muscle injuries [18, 53], no expert consensus was achieved for LARGP. In round 1, 56.5% of respondents (13/23) allows pain in palpation, while 43.5% (10/23) requires a complete absence of symptoms.

A total of 71.9% of experts uses PROMs but only 59.4% of them uses it as criteria in RTP. The PROMs most used by clinicians and researchers (91.3-21/23) is HAGOS [54].

Intersegmental Control analysis seems to be useful in managing groin pain [8]. Even if for this category no agreement was established, intersegmental control is used by 71.9% of sample. In particular, 78.3% of respondents (18/23) use single leg squat as a test to assess motor control.

Analysis of flexibility did not reach the consensus in its utilisation. Nevertheless, evidence highlights the importance of getting total hip range of motion to avoid recurrence episodes of groin pain [35].

Even if training load (TL) cannot be considered a valid tool to assess injury risk [55], as reported by Cummins et al. [56], load management could represent a helpful tool to manage RTP progression. However, in the present Delphi study neither internal nor external load parameters reached consensus.

### Discussion of Limitations

The over-representation of some geographical regions, working setting and specific expertise based on different healthcare profession could have introduced unintended bias, as well as the inclusion of experts with knowledge of the English language (required to understand the survey).

## Conclusion

Our research showed an agreement among experts on 4 out of 9 sections. As suggested by our expert panel, RTP framework is a complex process composed of several decision-modifiers, however, these findings could be a useful practical tool (Fig. 2) for clinicians in the “first-step” planning of RTP physical aspects assessment. Nevertheless, it would be desirable to establish a more solid and broad experts’ consensus, including assessment of other items, psychosocial factors and a wider and heterogeneous expert’s cohort.

## Supplementary Information

**Additional file 1**. Complete/extensive tables with expert panel answers in the three rounds.

## Data Availability

The Delphi rounds questions and datasets used and analysed during the current study are available from the corresponding author on reasonable request. Data include round 1, round 2 and round 3 Delphi questionnaire results and further demographic data.

## Abbreviations

LARGP: Longstanding adductor-related groin pain
RTP: Return to play
HAGOS: Hip and Groin Outcome Score
TL: Training load
IC: Intersegmental control
PROMs: Patient-reported outcome measures
COD: Change of direction
StARRT: Strategic assessment of risk and risk tolerance

## References

[1] Falvey EC, Franklyn-Miller A, McCrory PR (2009). The groin triangle: a patho-anatomical approach to the diagnosis of chronic groin pain in athletes. Br J Sports Med.

[2] Joyce D, Lewindon D. Sports injury prevention and rehabilitation: integrating medicine and science for performance solutions [Internet]. 1st edn. Routledge; 2015. Available from: https://www.taylorfrancis.com/books/9780203066485.

[3] Ekstrand J, Krutsch W, Spreco A, van Zoest W, Roberts C, Meyer T (2020). Time before return to play for the most common injuries in professional football: a 16-year follow-up of the UEFA Elite Club Injury Study. Br J Sports Med.

[4] Orchard J, Seward H (2002). Epidemiology of injuries in the Australian Football League, seasons 1997–2000. Br J Sports Med.

[5] Emery CA, Meeuwisse WH, Powell JW (1999). Groin and abdominal strain injuries in the National Hockey League. Clin J Sport Med.

[6] Brooks JHM, Fuller CW, Kemp SPT, Reddin DB (2005). Epidemiology of injuries in English professional rugby union: part 2 training Injuries. Br J Sports Med.

[7] Weir A, Brukner P, Delahunt E, Ekstrand J, Griffin D, Khan KM (2015). Doha agreement meeting on terminology and definitions in groin pain in athletes. Br J Sports Med.

[8] King E, Franklyn-Miller A, Richter C, O’Reilly E, Doolan M, Moran K (2018). Clinical and biomechanical outcomes of rehabilitation targeting intersegmental control in athletic groin pain: prospective cohort of 205 patients. Br J Sports Med.

[9] Daniels KAJ, King E, Richter C, Falvey É, Franklyn-Miller A (2020). Changes in the kinetics and kinematics of a reactive cut maneuver after successful athletic groin pain rehabilitation. Scand J Med Sci Sports.

[10] O’Connor D (2004). Groin injuries in professional rugby league players: a prospective study. J Sports Sci.

[11] Bradshaw CJ, Bundy M, Falvey E (2008). The diagnosis of longstanding groin pain: a prospective clinical cohort study. Br J Sports Med.

[12] Shrier I (2015). Strategic Assessment of Risk and Risk Tolerance (StARRT) framework for return-to-play decision-making. Br J Sports Med.

[13] Creighton DW, Shrier I, Shultz R, Meeuwisse WH, Matheson GO (2010). Return-to-play in sport: a decision-based model. Clin J Sport Med.

[14] Ardern CL, Glasgow P, Schneiders A, Witvrouw E, Clarsen B, Cools A (2016). 2016 Consensus statement on return to sport from the First World Congress in Sports Physical Therapy. Bern Br J Sports Med.

[15] Hasson F, Keeney S, McKenna H (2000). Research guidelines for the Delphi survey technique. J Adv Nurs.

[16] Lynn MR, Layman EL, Englebardt SP (1998). Nursing administration research priorities: a national Delphi study. J Nurs Adm.

[17] McKenna HP (1994). The Delphi technique: a worthwhile research approach for nursing?. J Adv Nurs.

[18] van der Horst N, Backx F, Goedhart EA, Huisstede BM (2017). HIPS-Delphi Group: return to play after hamstring injuries in football (soccer): a worldwide Delphi procedure regarding definition, medical criteria and decision-making. Br J Sports Med.

[19] McCall A, Pruna R, Van der Horst N, Dupont G, Buchheit M, Coutts AJ (2020). Exercise-based strategies to prevent muscle injury in male elite footballers: an expert-led delphi survey of 21 practitioners belonging to 18 teams from the big-5 European Leagues. Sports Med.

[20] Zambaldi M, Beasley I, Rushton A (2017). Return to play criteria after hamstring muscle injury in professional football: a Delphi consensus study. Br J Sports Med.

[21] Weir A, Hölmich P, Schache AG, Delahunt E, de Vos R-J (2015). Terminology and definitions on groin pain in athletes: building agreement using a short Delphi method. Br J Sports Med.

[22] Donaldson A, Cook J, Gabbe B, Lloyd DG, Young W, Finch CF (2015). Bridging the gap between content and context: establishing expert consensus on the content of an exercise training program to prevent lower-limb injuries. Clin J Sport Med.

[23] Kleynen M, Braun SM, Bleijlevens MH, Lexis MA, Rasquin SM, Halfens J (2014). Using a Delphi technique to seek consensus regarding definitions, descriptions and classification of terms related to implicit and explicit forms of motor learning. PLoS ONE.

[24] Sheu S-J, Wei I-L, Chen C-H, Yu S, Tang F-I (2009). Using snowball sampling method with nurses to understand medication administration errors. J Clin Nurs.

[25] Bisciotti GN, Volpi P, Alberti G, Aprato A, Artina M, Auci A (2019). Italian consensus statement (2020) on return to play after lower limb muscle injury in football (soccer). BMJ Open Sport Exerc Med.

[26] Joyner HS, Smith D (2015). Using Delphi surveying techniques to gather input from non-academics for development of a modern dairy manufacturing curriculum. J Food Sci Educ.

[27] van Alphen A, Halfens R, Hasman A, Imbos T (1994). Likert or Rasch? Nothing is more applicable than good theory. J Adv Nurs.

[28] Côté J, Salmela JH, Baria A, Russell SJ (1993). Organizing and interpreting unstructured qualitative data. Sport Psychol.

[29] Verhagen AP, de Vet HC, de Bie RA, Kessels AG, Boers M, Bouter LM (1998). The Delphi list: a criteria list for quality assessment of randomized clinical trials for conducting systematic reviews developed by Delphi consensus. J Clin Epidemiol.

[30] Huisstede BMA, Hoogvliet P, Coert JH, Fridén J (2014). European HANDGUIDE Group: multidisciplinary consensus guideline for managing trigger finger: results from the European HANDGUIDE Study. Phys Ther.

[31] von der Gracht HA (2012). Consensus measurement in Delphi studies: review and implications for future quality assurance. Technol Forecast Soc Chang.

[32] Kloskowska P, Morrissey D, Small C, Malliaras P, Barton C (2016). Movement patterns and muscular function before and after onset of sports-related groin pain: a systematic review with meta-analysis. Sports Med.

[33] Mosler AB, Weir A, Serner A, Agricola R, Eirale C, Farooq A (2018). Musculoskeletal screening tests and bony hip morphology cannot identify male professional soccer players at risk of groin injuries: a 2-year prospective cohort study. Am J Sports Med.

[34] Serner A, Weir A, Tol JL, Thorborg K, Lanzinger S, Otten R (2020). Return to sport after criteria-based rehabilitation of acute adductor injuries in male athletes: a prospective cohort study. Orthop J Sports Med.

[35] Nevin F, Delahunt E (2014). Adductor squeeze test values and hip joint range of motion in Gaelic football athletes with longstanding groin pain. J Sci Med Sport.

[36] Thorborg K, Branci S, Nielsen MP, Tang L, Nielsen MB, Hölmich P (2014). Eccentric and isometric hip adduction strength in male soccer players with and without adductor-related groin pain: an assessor-blinded comparison. Orthop J Sports Med.

[37] Ishøi L, Thorborg K. Copenhagen adduction exercise can increase eccentric strength and mitigate the risk of groin problems: but how much is enough! Br J Sports Med. 2021. 10.1136/bjsports-2020-103564 33627335

[38] Harøy J, Clarsen B, Wiger EG, Øyen MG, Serner A, Thorborg K (2019). The adductor strengthening programme prevents groin problems among male football players: a cluster-randomised controlled trial. Br J Sports Med.

[39] Mosler AB, Agricola R, Weir A, Hölmich P, Crossley KM (2015). Which factors differentiate athletes with hip/groin pain from those without? A systematic review with meta-analysis. Br J Sports Med.

[40] Gore SJ, Franklyn-Miller A, Richter C, Falvey EC, King E, Moran K (2018). Is stiffness related to athletic groin pain?. Scand J Med Sci Sports.

[41] Thorborg K, Reiman MP, Weir A, Kemp JL, Serner A, Mosler AB (2018). Clinical examination, diagnostic imaging, and testing of athletes with groin pain: an evidence-based approach to effective management. J Orthop Sports Phys Ther.

[42] Serner A, Weir A, Tol JL, Thorborg K, Yamashiro E, Guermazi A (2020). Associations between initial clinical examination and imaging findings and return-to-sport in male athletes with acute adductor injuries: a prospective cohort study. Am J Sports Med.

[43] Branci S, Thorborg K, Nielsen MB, Hölmich P (2013). Radiological findings in symphyseal and adductor-related groin pain in athletes: a critical review of the literature. Br J Sports Med.

[44] Franklyn-Miller A, Richter C, King E, Gore S, Moran K, Strike S (2017). Athletic groin pain (part 2): a prospective cohort study on the biomechanical evaluation of change of direction identifies three clusters of movement patterns. Br J Sports Med.

[45] Marshall BM, Franklyn-Miller AD, Moran KA, King EA, Strike SC, Falvey ÉC (2016). Can a single-leg squat provide insight into movement control and loading during dynamic sporting actions in patients with athletic groin pain?. J Sport Rehabil.

[46] Gabbe BJ, Finch CF, Wajswelner H, Bennell KL (2004). Predictors of lower extremity injuries at the community level of Australian football. Clin J Sport Med.

[47] Chaudhari AMW, Jamison ST, McNally MP, Pan X, Schmitt LC (2014). Hip adductor activations during run-to-cut manoeuvres in compression shorts: implications for return to sport after groin injury. J Sports Sci.

[48] Welch N, Richter C, Moran K, Franklyn-Miller A (2020). Rehabilitation interventions need more than methodological standardisation: an individualised approach. BMJ Open Sport Exerc Med.

[49] Rivadulla AR, Gore S, Preatoni E, Richter C (2020). Athletic groin pain patients and healthy athletes demonstrate consistency in their movement strategy selection when performing multiple repetitions of a change of direction test. J Sci Med Sport.

[50] Serner A, Hölmich P, Tol JL, Thorborg K, Lanzinger S, Otten R (2021). Progression of strength, flexibility, and palpation pain during rehabilitation of athletes with acute adductor injuries: a prospective cohort study. J Orthop Sports Phys Ther.

[51] Kerbel YE, Smith CM, Prodromo JP, Nzeogu MI, Mulcahey MK (2018). Epidemiology of hip and groin injuries in collegiate athletes in the united states. Orthop J Sports Med.

[52] Buckthorpe M, Della Villa F, Della Villa S, Roi GS (2019). On-field rehabilitation part 2: a 5-stage program for the soccer player focused on linear movements, multidirectional movements, soccer-specific skills, soccer-specific movements, and modified practice. J Orthop Sports Phys Ther.

[53] De Vos R-J, Reurink G, Goudswaard G-J, Moen MH, Weir A, Tol JL (2014). Clinical findings just after return to play predict hamstring re-injury, but baseline MRI findings do not. Br J Sports Med.

[54] Thorborg K, Hölmich P, Christensen R, Petersen J, Roos EM (2011). The Copenhagen Hip and Groin Outcome Score (HAGOS): development and validation according to the COSMIN checklist. Br J Sports Med.

[55] Impellizzeri FM, Woodcock S, Coutts AJ, Fanchini M, McCall A, Vigotsky AD (2021). What role do chronic workloads play in the acute to chronic workload ratio? Time to dismiss ACWR and its underlying theory. Sports Med.

[56] Cummins C, Orr R, O’Connor H, West C (2013). Global positioning systems (GPS) and microtechnology sensors in team sports: a systematic review. Sports Med.

