# Supplementary material for: Return to Play in Long-Standing Adductor-Related Groin Pain: A Delphi Study Among Experts
Source: Sports Med Open. 2022 Jan 18;8:11. doi: 10.1186/s40798-021-00400-z (PMC8766680; doi:10.1186/s40798-021-00400-z)
Supplement: Supplementary file 1 — Additional file 1. Complete/extensive tables with expert panel answers in the three rounds. [file 40798_2021_400_MOESM1_ESM.docx]

| TABLE 1. Round 1 Questionnaire Results. | | | | | | | |  |
| --- | --- | --- | --- | --- | --- | --- | --- | --- |
| Section | **Item** | | **Consensus** | | **Percentage (%)** | | |  |
| Palpation | Use of palpation in RTP process | | + | | | 78.1 | |  |
|  | Presence of pain during palpation | | + | | | 92.0 | |  |
|  | Allow RTP with pain in palpation | | NC | | | 56.5 | |  |
|  | Other parameters considered^a^ | |  | | |  | |  |
|  |  | Pain parameters | | 7 respondents^b^ | | | | |
|  |  | Localization | | 5 respondents^b^ | | | | |
|  |  | Tightness | | 4 respondents^b^ | | | | |
|  |  | Type of tissue | | 4 respondents^b^ | | | | |
|  |  | Irritability | | 1 respondent | | | | |
| Flexibility | Flexibility analysis in RTP process | | NC | | | 62.5 | |  |
|  | Test/s used^a^ | |  | | |  | |  |
|  |  | Internal Rotation - knee/hip bended supine | | 13 respondents | | | | |
|  |  | Internal Rotation - knee bended prone | | 4 respondents | | | | |
|  |  | External Rotation - hip/knee bended supine | | 11 respondents | | | | |
|  |  | External Rotation - knee bended prone | | 3 respondents | | | | |
|  |  | Adductor test | | 12 respondents | | | | |
|  |  | Thomas test | | 11 respondents | | | | |
|  |  | Other tests suggested by experts | | 1 respondents (each) | | | | |
|  | Presence of pain in flexibility tests | | NC | | | 85.0 | |  |
|  | Allow RTP with pain in flexibility tests | | NC | | | 88.2 | |  |
|  | Others parameters considered^a^ | |  | | |  | |  |
|  | Range of motion | | 8 respondents | | | | |  |
|  | Side-to-side symmetry | | 3 respondents | | | | |  |
|  | End Feel | | 2 respondents | | | | |  |
| Strength | Strength analysis in RTP process | | + | | | 96.9 | |  |
|  | Hip muscle groups (type of strength) | |  | | |  | |  |
|  |  | Adductors (Ecc/Iso/Conc) | | + Eccentric ;  + Isometric | | | 87.1/74.2/38.7 | |
|  |  | Abductors (Ecc/Iso/Conc) | | NC | | | 51.6/51.6/  12.9 | |
|  |  | Extensors (Ecc/Iso/Conc) | | NC | | | 38.7/9.7/  16.1 | |
|  |  | Flexors (Ecc/Iso/Conc) | | NC | | | 54.8/32.3/  29.0 | |
|  |  | Internal Rotators (Ecc/Iso/Conc) | | NC | | | 38.7/9.7/  22.6 | |
|  |  | External Rotators (Ecc/Iso/Conc) | | NC | | | 38.7/6.5/  19.4 | |
|  | Strength assessment of other muscle groups | | + | | | 71.0 | |  |
|  | Which other areas?^a^ | |  | | |  | |  |
|  |  | Trunk group | | 18 respondents^b^ | | | | |
|  |  | Knee group | | 9 respondents^b^ | | | | |
|  |  | Calf complex group | | 7 respondents^b^ | | | | |
|  |  | Pubis group | | 2 respondents | | | | |
|  | Presence of pain in strength tests | | + | | | 96.8 | |  |
|  | Allow RTP with pain in strength tests | | - | | | 70.0 | |  |
|  | Other parameters considered | |  | | |  | |  |
|  |  | Pain parameters (location, grading) | | 10 respondents^b^ | | | | |
|  |  | Side-to-side symmetry | | 8 respondents^b^ | | | | |
|  |  | Ratios | | 8 respondents^b^ | | | | |
|  |  | Baseline data | | 7 respondents^b^ | | | | |
|  |  | Patient feedback | | 5 respondents | | | | |
|  |  | Rate of force development | | 3 respondents | | | | |
|  |  | Rate of contractions | | 1 respondent | | | | |
| PROMs | Use of PROMs | | + | | | 71.9 | |  |
|  | Which PROMs?^a^ | |  | | |  | |  |
|  |  | Hip And Groin Outcome Score (HAGOS) | | 21 respondents^b^ | | | | |
|  |  | Visual Analog Scale (VAS) | | 2 respondents^b^ | | | | |
|  |  | Internal PROMs | | 2 respondents^b^ | | | | |
|  |  | Other PROMs suggested by experts | | 1 respondent (each) | | | | |
| Imaging | Use of imaging in RTP process | | - | | | 75.0 | |  |
|  | Imaging methods used^a^ | |  | | |  | |  |
|  |  | Magnetic resonance | | 7 respondents | | | | |
|  |  | Ultrasound | | 3 respondents | | | | |
|  |  | Computer tomography | | 1 respondent | | | | |
|  |  | Radiography | | 4 respondents | | | | |
| Intersegmental Control (IC) | IC tasks analysis in RTP process | | + | | | 71.9 | |  |
|  | Which IC tasks?^a^ | |  | | |  | |  |
|  |  | Single leg squat | | 18 respondents^b^ | | | | |
|  |  | Squat | | 15 respondents^b^ | | | | |
|  |  | Lunge | | 13 respondents^b^ | | | | |
|  |  | Drop Jump | | 9 respondents | | | | |
|  |  | Deadlift | | 6 respondents | | | | |
|  |  | Other tasks suggested by experts | | 1 respondent (each) | | | | |
|  | Presence of pain in IC tasks | | + | | | 82.6 | |  |
|  | Allow RTP with pain in IC tasks | | - | | | 84.2 | |  |
|  | Other parameters considered^a^ | |  | | |  | |  |
|  |  | Quality of movement | | 10 respondents^b^ | | | | |
|  |  | Side-to-side symmetry | | 3 respondents^b^ | | | | |
|  |  | Pain parameters (location, grading) | | 2 respondents^b^ | | | | |
|  |  | Baseline values | | 1 respondent | | | | |
|  |  | Joint velocity | | 1 respondent | | | | |
| Performance Tests | Performance tests analysis in RTP process | | + | | | 78.1 | |  |
|  | Which performance tests?^a^ | |  | | |  | |  |
|  |  | Planned/unplanned COD (45-90-180) | | 18 respondents^b^ | | | | |
|  |  | T-test | | 17 respondents^b^ | | | | |
|  |  | Illinois test | | 13 respondents^b^ | | | | |
|  |  | Shuttle run | | 6 respondents | | | | |
|  |  | Shuffle run | | 5 respondents | | | | |
|  |  | Other performance tests suggested by experts | | 1 respondent (each) | | | | |
|  | Presence of pain in performance tests | | + | | | 100.0 | |  |
|  | Allow RTP with pain in performance tests | | - | | | 80.0 | |  |
|  | Other parameters considered^a^ | |  | | |  | |  |
|  |  | Performance/intensity | | 8 respondents^b^ | | | | |
|  |  | Grading of pain | | 6 respondents^b^ | | | | |
|  |  | Movement control | | 4 respondents^b^ | | | | |
|  |  | Baseline values | | 3 respondents | | | | |
|  |  | Others parameters suggested by experts | | 1 respondent (each) | | | | |
| Sport-Specific Skills | Sport-specific skills evaluation in RTP process | | + | | | 87.5 | |  |
|  | Presence of pain in Sport-Specific skills | | + | | | 89.3 | |  |
|  | Allow RTP with pain in sport-specific skills execution | | - | | | 84.0 | |  |
|  | Other parameters considered^a^ | |  | | |  | |  |
|  |  | Pain parameters (location, grading) | | 7 respondents^b^ | | | | |
|  |  | Performance/intensity | | 5 respondents^b^ | | | | |
|  |  | Specific tests | | 3 respondents^b^ | | | | |
|  |  | Quality of movement | | 3 respondents^b^ | | | | |
|  |  | Athlete feedback | | 3 respondents^b^ | | | | |
|  |  | Baseline values | | 2 respondents | | | | |
| Training Load |  | |  | | |  | |  |
|  | Internal Load monitoring in RTP process | | NC | | | 56.3 | |  |
|  | Parameters considered^a^ | | NC | | |  | |  |
|  |  | Rated Perceived Exertion (RPE) scale | | 15 respondents | | | | |
|  |  | Heart rate | | 2 respondents | | | | |
|  |  | Others parameters suggested by experts | | 1 respondents (each) | | | | |
|  | External Load monitoring in RTP process | | NC | | | 59.4 | |  |
|  | Parameters considered^a^ | |  | | |  | |  |
|  |  | N° of full training session | | 13 respondents | | | | |
|  |  | GPS tracking | | 7 respondents | | | | |
|  |  | Performance tests | | 9 respondents | | | | |
|  |  | Other parameters suggested by experts | | 1 respondent (each) | | | | |
| (+) *Positive consensus;* (-) *Negative consensus;* NC,*No consensus;*  ^a^,*Open-ended question;* ^b^,*Top 3 ranked preferences for open-ended question coded independently by 2 researchers;*  *Abbreviations: Ecc,Eccentric; Iso,Isometric; Conc,Concentric; PROMs, Patient Reported Outcome Measures; CoD, Changes of Direction* | | | | | | | |  |

| TABLE 2. Round 2 Questionnaire Results. | | | | | |
| --- | --- | --- | --- | --- | --- |
| Section | **Item** | | **Consensus** | **Percentage(%)** | |
| Palpation | Use as a criterion in RTP process | | NC | 68.8 | |
| Strength | Use as criterion in RTP process | | + | 93.8 | |
|  | Tests to evaluate hip Adductors' Isometric Strength | |  |  | |
|  |  | Squeeze test 0° | NC | 66.7 | |
|  |  | Squeeze test 45° | NC | 60.0 | |
|  |  | Squeeze test 90° | NC | 20.0 | |
|  |  | Copenaghen five-second squeeze test | NC | 43.3 | |
|  |  | Supine position | NC | 46.7 | |
|  |  | Side position | NC | 26.7 | |
|  |  | Standing position | NC | 10.0 | |
|  |  | Other tests | NC | 3.3 (each) | |
|  | Tests to evaluate hip Adductors' Eccentric Strength | |  |  | |
|  |  | Supine position | NC | 33.3 | |
|  |  | Standing position | NC | 13.3 | |
|  |  | Side position | NC | 53.3 | |
|  |  | Copenaghen adduction exercise | NC | 40.0 | |
|  |  | Other tests | NC | 3.3 (each) | |
|  | Analysis of strength in other muscle groups | | NC | 66.7 | |
|  | Which muscle groups? | |  |  | |
|  |  | Trunk Flexors | NC | 90.0 | |
|  |  | Knee flexor | NC | 55.0 | |
|  |  | Trunk Extensor | NC | 45.0 | |
|  |  | Knee extensor | NC | 40.0 | |
|  |  | Calf complex | NC | 35.0 | |
|  |  | Trunk side-bending and rotators | NC | 11.0 | |
|  | Others parameters considered (except pain) | |  |  | |
|  |  | Side-to-Side Symmetry | + | 80.0 | |
|  |  | Add/Abd strength ratio | NC | 63.3 | |
|  |  | Baseline data pre-injury | NC | 56.7 | |
|  |  | Normative data presented in literature | NC | 43.3 | |
|  |  | Other parameters | NC | 3.3 (each) | |
| PROMs | Use as a criterion in RTP process | | NC | 59.4 | |
|  | Does expert use a minimum score for HAGOS? | | NC | 52.6 | |
|  | Which minimum score? | |  |  | |
|  |  | 70 | NC | 30.0 | |
|  |  | 76 | NC | 10.0 | |
|  |  | 80 | NC | 30.0 | |
|  |  | 90 | NC | 30.0 | |
|  | HAGOS Sub-scales used | |  |  | |
|  |  | All | NC | 62.5 | |
|  |  | Symptoms | NC | 25.0 | |
|  |  | Pain | NC | 31.3 | |
|  |  | Physical function, daily living | NC | 0.0 | |
|  |  | Function, sports and recreational activity | NC | 31.3 | |
|  |  | Participation in physical activity | NC | 12.5 | |
|  |  | Quality of life | NC | 12.5 | |
| Imaging | Use as a criterion in RTP process | | - | 78.1 | |
| Intersegmental Control (IC) | Use as a criterion in RTP process | | NC | 34.3 | |
|  | Others parameters considered (except pain) | |  |  | |
|  |  | Side-to-side symmetry | NC | 90.9 | |
|  |  | Athlete feedback | NC | 63.6 | |
|  |  | Quality of movement (video analysis) | NC | 54.5 | |
|  |  | Quality of movement | NC | 81.8 | |
|  | Segments analyzed | |  |  | |
|  |  | Thorax on pelvis | NC | 72.7 | |
|  |  | Pelvis on femur | NC | 81.8 | |
|  |  | Femur on tibia | NC | 63.6 | |
|  |  | Tibia on foot | NC | 54.5 | |
| Performance tests | Use as a criterion in RTP process | | + | 90.6 | |
|  | Others parameters considered (except pain) | |  |  | |
|  |  | Athlete feedback | + | 82.8 | |
|  |  | Comparing execution time with controlateral | NC | 58.6 | |
|  |  | Comparing execution time with pre-injury data | NC | 41.4 | |
|  |  | Quality of movement | NC | 62.1 | |
|  |  | Other parameters | NC | 3.4 (each) | |
| Sport-Specific Skills | Use as a criterion in RTP process | | + | 90.6 | |
|  | Others parameters considered (except pain) | |  |  | |
|  |  | Athlete feedback | + | 86.2 | |
|  |  | Performance in skills execution | + | 75.9 | |
|  |  | Quality of movement | + | 72.4 | |
|  |  | Other parameters | NC | 3.4 (each) | |
| (+) *Positive consensus;* (-) *Negative consensus;* NC, *No consensus*  *Abbrevations: PROMs, Patient Reported Outcome Measures* | | | | |  |
|  | | | | | |

| TABLE 3. Round 3 Questionnaire (Final Agreement) Results. | | | | | | | | |
| --- | --- | --- | --- | --- | --- | --- | --- | --- |
|  | 1 - Strongly Disagree (%) | 2 – Disagree (%) | 3 – Neutral  (%) | 4 –Agree (%) | 5 - Strongly Agree (%) | Mean±SD | Coefficient of Variation (%) | % of agreement |
| Strength is a criterion when evaluating RTP | 0.0 | 0.0 | 3.1 | 34.4 | 62.5 | 4.6±0.6 | 12.2 | 96.9 |
| Analysis of isometric strength of hip adductors as a criterion when evaluating RTP | 0.0 | 9.4 | 15.6 | 50.0 | 25.0 | 3.9±0.9 | 22.9 | 75.0 |
| Analysis of eccentric strength of hip adductors as a criterion when evaluating RTP | 0.0 | 3.1 | 12.5 | 46.9 | 37.5 | 4.2±0.8 | 18.6 | 84.4 |
| As a criterion, Athletes have to be pain-free in strength assessment tests when evaluating RTP | 0.0 | 15.6 | 18.8 | 37.5 | 28.1 | 3.8±1.0 | 27.5 | 65.6 |
| Analysis of side-to-side symmetry in strength tests as a criterion when evaluating RTP | 0.0 | 3.1 | 9.4 | 56.3 | 31.3 | 4.2±0.7 | 17.4 | 87.6 |
| Use of imaging methods is not a criterion when evaluating RTP | 3.1 | 3.1 | 0.0 | 43.8 | 50.0 | 4.3±0.9 | 20.8 | 93.8 |
| Use of performance tests is a criterion when evaluating RTP | 0.0 | 0.0 | 6.3 | 31.3 | 62.5 | 4.6±0.6 | 13.6 | 93.8 |
| Change of Direction analysis is a criterion when evaluating RTP | 0.0 | 0.0 | 3.1 | 37.5 | 59.4 | 4.6±0.6 | 12.4 | 96.9 |
| As a criterion, Athletes have to be pain-free in performance tests when evaluating RTP | 0.0 | 6.3 | 15.6 | 50.0 | 28.1 | 4.0±0.8 | 21.1 | 78.1 |
| Athlete feedback during performance tests is a criterion when evaluating RTP | 0.0 | 0.0 | 6.3 | 43.8 | 50.0 | 4.4±0.6 | 13.9 | 93.8 |
| Analysis of sport-specific skills is a criterion when evaluating RTP | 0.0 | 0.0 | 3.1 | 34.4 | 62.5 | 4.6±0.6 | 12.2 | 96.9 |
| As a criterion, Athletes have to be pain-free in sport-specific skills execution when evaluating RTP | 0.0 | 6.3 | 18.8 | 46.9 | 28.1 | 4.0±0.9 | 21.7 | 75.0 |
| Athlete feedback during sport-specific skills execution is a criterion when evaluating RTP | 0.0 | 0.0 | 3.1 | 46.9 | 50.0 | 4.5±0.6 | 12.7 | 96.9 |
| Analysis of performance in sport-specific skills execution is a criterion when evaluating RTP | 0.0 | 3.1 | 31.3 | 53.1 | 12.5 | 3.8±0.7 | 19.2 | 65.6 |
| Analysis of quality of movement during sport-specific skills execution is a criterion when evaluating RTP | 0.0 | 21.9 | 12.5 | 46.9 | 18.8 | 3.6±1.0 | 28.7 | 65.7 |
| *Statistics Kendall W*= 0,03 |  |  |  |  |  |  |  |  |
